# Mental health outcomes after SARS-CoV-2 vaccination in the United States: A national cross-sectional study

**DOI:** 10.1016/j.jad.2021.10.134

**Published:** 2022-02-01

**Authors:** Shanquan Chen, Athina R. Aruldass, Rudolf N. Cardinal

**Affiliations:** a Department of Psychiatry, University of Cambridge, Cambridge CB2 0SZ, United Kingdom; b Cambridgeshire and Peterborough NHS Foundation Trust, CB21 5EF, United Kingdom

**Keywords:** SARS-CoV-2 vaccination, Anxiety, Depression, United States

## Abstract

**Background:**

Worsening of anxiety and depressive symptoms have been widely described during the COVID-19 pandemic. It can be hypothesized that vaccination could link to reduced symptoms of anxiety and/or depression. However, to date, no study has assessed this. This study aims to examine anxiety and depressive symptoms after vaccination in US adults, meanwhile test sociodemographic disparities in these outcomes.

**Methods:**

Data from the January 6-June 7 2021, cross-sectional Household Pulse Survey were analyzed. Using survey-weighted logistic regression, we assessed the relationships between SARS-CoV-2 vaccination and anxiety and/or depressive symptoms, both on overall and sociodemographic subgroups. We controlled for a variety of potential socioeconomic and demographic confounding factors.

**Results:**

Of the 453,167 participants studied, 52.2% of the participants had received the COVID-19 vaccine, and 26.5% and 20.3% of the participants reported anxiety and depression, respectively. Compared to those not vaccinated, the vaccinated participants had a 13% lower odds of anxiety (adjusted odds ratio [AOR] = 0.85, 95%CI 0.83–0.90) and 17% lower odds of depression (AOR = 0.83, 95%CI 0.79–0.85). Disparities on the above associations were identified in age, marital status, education level, ethnic/race, and income level, but not on gender.

**Limitations:**

The causal inference was not able to be investigated due to the cross-sectional study design.

**Conclusion:**

Being vaccinated for SARS-CoV-2 was associated with lower odds of anxiety and/or depressive symptoms. While those more middle-aged or more affluent, were more likely to show these negative associations, the contrary was observed in ethnic minorities and those with lower educational attainment. More strategic and demography-sensitive public health communications could perhaps temper these issues.

## Highlights

• Being vaccinated for SARS-CoV-2 was associated with lower odds of anxiety and/or depressive symptoms.
• Those more middle-aged or more affluent were more likely to show a stronger association between SARS-CoV-2 vaccination and reduced anxiety and/or depressive symptoms.
• Vaccination among ethnic minorities and those with lower educational attainment associated with additional higher odds of anxiety or depression.

## Introduction

The ongoing COVID-19 pandemic urgently needs the achievement of global SARS-CoV-2 vaccination coverage. Given the considerable increase in anxiety and depressive symptoms caused by the stress sourcing from the COVID-19 pandemic (Vahratian et al., 2021), it's reasonable to hypothesize that the vaccination could link to reduced symptoms of anxiety and/or depression. However, recently reported clusters of anxiety-related events after administration of COVID-19 vaccine in five mass vaccination sites in the US reminded that the vaccination could conversely worse vaccinees’ mental problems (Hause et al., 2021). These events reported in the US violated the above hypothesis, or implied that there may be subgroup disparities in the association between SARS-CoV-2 vaccination and reduced symptoms of anxiety and/or depression. To date, no study has assessed this. In this study, we aim to examine anxiety and depressive symptoms after vaccination in US adults, meanwhile test sociodemographic disparities in these outcomes.

Extant studies have reported on the heightened vulnerability of those with mental illness to coronavirus (Mazereel et al., 2021), vaccine hesitancy (Khubchandani et al., 2021) and vaccine efficacy amongst this subgroup (Mazereel et al., 2021). The present study will contribute to our knowledge on SARS-CoV-2 vaccination and mental health, and help the improvement of policy strategy of SARS-CoV-2 vaccination.

## Methods

### Study design and participants

This study used data from the Household Pulse Survey (HPS), a nationally representative survey of adults measuring the impact of the COVID-19 pandemic, conducted by the U.S. Census Bureau in partnership with the Centers for Disease Control and Prevention. The HPS used the US Census Bureau's Master Address File as the source of sampled housing units. The sample design was a systematic sample of all eligible housing units, with adjustments applied to the sampling intervals to select a large enough sample to create representative estimates at the national, state, and metropolitan area levels. Technical details are available on the Census Bureau website (Hause et al., 2021). HPS was administered online and gathered demographic, social, economic and health information, week of the interview, and state of origin. We utilized HPS data spanning January 6 through June 7 2021, which also includes COVID-19 vaccine-related queries.

The data are publicly available. The use of secondary de-identified data making this study exempt from institutional review board review. This study follows the Strengthening the Reporting of Observational Studies in Epidemiology (STROBE) reporting guideline (STROBE checklist for cohort studies, 2007).

### Measures

Vaccination status was qualitatively assessed by the question “Have you received a COVID-19 vaccine?” with the response yes or no. Questions on mental health symptoms were based on the validated two-item Generalized Anxiety Disorder for anxiety and two-item Patient Health Questionnaire for depression. Both scores range from 0 to 6, and a score ≥3 on each scale indicates a high probability of the disorder (Kroenke et al., 2003; Kroenke et al., 2007).

### Statistical analyze

We report categorical variables as number (percentage), and continuous variables mean (standard deviation). Survey weighting per wave was used to account for sampling design (including the unequal probability of selection, clustering, and stratification).

To estimate the association between vaccination and mental health outcomes, we fitted weighted logistic models with anxiety or depression as the dependent variables, and vaccination status as the predictor, controlling for covariates, including age, gender, race/ethnicity, educational attainment, marital status, household income, history of COVID-19 infection, week of the interview, and state of origin. To estimate subgroup disparities in the above association, we fitted similar models but adding an interaction between interested subgroup factors and vaccination status.

We also repeated the above analyses for each state.

All analyses were performed using R, version 3.6.0. We report two-sided *P* values and 95% confidence intervals (CIs) throughout. *P* < 0.05 was considered to be statistically significant.

## Results

Of the 453,167 participants studied, most identified as women (59.7%), White (78.3%), and were aged between 45 and 65 (41.7%). 52.2% of the participants had received the COVID-19 vaccine. 26.5% of the participants reported anxiety and 20.3% reported depression (Table 1).

**Table 1:** Characteristics of Participants. Data are from the household pulse survey among adults 18 or older spanning January 6 through June 7 2021.

Characteristic	No. (%) of participants
**Gender(= Female)**	270 350(59.7)
**Age**	
18–44	134 518(29.7)
45–65	189 125(41.7)
65+	129 524(28.6)
**Race/ethnicity**	
White	354 914(78.3)
Black	31 443(6.9)
Asian	17 996(4)
Hispanic	34 402(7.6)
Other	14 412(3.2)
**Education attained**	
Less than high school	2 233(0.5)
Some high school	4 937(1.1)
High school graduate or equivalent	47 886(10.6)
Some college, but degree not received or is in progress	92 299(20.4)
Associate's degree	48 081(10.6)
Bachelor's degree	135 068(29.8)
Graduate degree	122 663(27.1)
**Marital Status**	
Married	270 527(59.7)
Never married	78 235(17.3)
Widowed/divorced/separated	104 405(23)
**Total household income before taxes**	
Less than $25,000	45 179(10)
$25,000 - $34,999	39 007(8.6)
$35,000 - $49,999	49 706(11)
$50,000 - $74,999	81 244(17.9)
$75,000 - $99,999	66 700(14.7)
$100,000 - $149,999	83 431(18.4)
$150,000 - $199,999	40 563(9)
$200,000 and above	47 337(10.4)
**History of doctor-confirmed COVID-19 infection**	48 076(10.6)
**Vaccine received**	236 757(52.2)
**Anxiety present**	119 883(26.5)
**Depression present**	91 964(20.3)

Compared to those not vaccinated, the vaccinated cohort had a 15% lower odds of anxiety (adjusted odds ratio [AOR] = 0.85, 95%CI 0.83–0.90) and 17% lower odds of depression (AOR = 0.83, 95%CI 0.79–0.85). These negative associations were stronger in participants aged ≥45, widowed/divorced/separated, and of higher income bracket (AORs < 1, *p* < 0.05) (Table 2).

**Table 2:** Association between SARS-CoV-2 vaccination and anxiety or depressive disorders, as well as sociodemographic disparities, January 6- June 7 2021,. Data are from the household pulse survey among adults 18 or older spanning January 6 through June 7 2021,. Covariates controlled for included age, gender, race/ethnicity, educational attainment, marital status, household income, history of COVID-19 infection, week of interview, and state of origin. (AOR, adjusted odds ratio; CI, confidence interval.).

**Characteristic**	**Anxiety(= TRUE)**		**Depression(= TRUE)**	
	AOR (95%CI)	*p*	AOR (95%CI)	*p*
**Main effect**				
Vaccine received (=No)	Reference	Reference	Reference	Reference
Vaccine received (=Yes)	0.85(0.83, 0.88)	<0.0001	0.83(0.79, 0.85)	<0.0001
**Subgroup disparity**				
**Age**				
Vaccine (=Yes) × age (=18–44)	Reference	Reference	Reference	Reference
Vaccine (=Yes) × age (=45–65)	0.85(0.81, 0.90)	< 0.0001	0.90(0.85, 0.97)	0.0024
Vaccine (=Yes) × age (=65+)	0.75(0.70, 0.81)	< 0.0001	0.74(0.68, 0.80)	<0.0001
**Gender**				
Vaccine (=Yes) × gender(=Male)	Reference	Reference	Reference	Reference
Vaccine (=Yes) × gender(=Female)	1.05(0.99, 1.11)	0.0814	1.02(0.96, 1.07)	0.5868
**Marital Status**				
Vaccine (=Yes) × marital status(=Married)	Reference	Reference	Reference	Reference
Vaccine (=Yes) × marital status(=Never married)	1.04(0.98, 1.12)	0.2213	1.01(0.94, 1.08)	0.8737
Vaccine (=Yes) × marital status(=Widowed/divorced/separated)	0.91(0.86, 0.98)	0.0079	0.93(0.87, 1.00)	0.0608
**Race/ethnicity**				
Vaccine (=Yes) × race/ethnicity (=White)	Reference	Reference	Reference	Reference
Vaccine (=Yes) × race/ethnicity (=Black)	1.23(1.12, 1.35)	<0.0001	1.09(0.99, 1.21)	0.0906
Vaccine (=Yes) × race/ethnicity (=Asian)	1.01(0.90, 1.14)	0.8381	1.02(0.90, 1.16)	0.7794
Vaccine (=Yes) × race/ethnicity (=Hispanic)	1.15(1.05, 1.26)	0.0017	1.27(1.15, 1.39)	<0.0001
Vaccine (=Yes) × race/ethnicity (=Other)	1.11(0.98, 1.26)	0.0989	1.03(0.90, 1.19)	0.662
**Education attained**				
Vaccine (=Yes) × education(=Less than high school)	1.45(1.08, 1.92)	0.0132	1.30(0.96, 1.75)	0.0927
Vaccine (=Yes) × education(=Some high school)	1.16(0.95, 1.42)	0.1427	1.27(1.03, 1.57)	0.0266
Vaccine (=Yes) × education(=High school graduate or equivalent)	1.05(0.98, 1.14)	0.1582	1.07(0.98, 1.16)	0.1121
Vaccine (=Yes) × education(=Some college, but degree Yet received or is in progress)	1.08(1.02, 1.15)	0.0115	1.12(1.04, 1.20)	0.0028
Vaccine (=Yes) × education(=Associate's degree)	1.08(1.00, 1.17)	0.0394	1.13(1.03, 1.22)	0.0076
Vaccine (=Yes) × education(=Bachelor's degree)	1.07(1.01, 1.14)	0.0165	1.09(1.03, 1.17)	0.0054
Vaccine (=Yes) × education(=Graduate degree)	Reference	Reference	Reference	Reference
**Total household income before taxes**				
Vaccine (=Yes) × household income(=Less than $25,000)	Reference	Reference	Reference	Reference
Vaccine (=Yes) × household income(=$25,000 - $34,999)	0.91(0.81, 1.02)	0.1119	0.91(0.81, 1.03)	0.1253
Vaccine (=Yes) × household income(=$35,000 - $49,999)	0.95(0.85, 1.06)	0.3533	0.90(0.80, 1.00)	0.0535
Vaccine (=Yes) × household income(=$50,000 - $74,999)	0.88(0.79, 0.96)	0.0065	0.92(0.84, 1.02)	0.1112
Vaccine (=Yes) × household income(=$75,000 - $99,999)	0.89(0.80, 0.98)	0.0242	0.89(0.79, 0.99)	0.0312
Vaccine (=Yes) × household income(=$100,000 - $149,999)	0.84(0.76, 0.92)	0.0003	0.81(0.73, 0.90)	0.0001
Vaccine (=Yes) × household income(=$150,000 - $199,999)	0.79(0.71, 0.89)	0.0001	0.84(0.73, 0.94)	0.0045
Vaccine (=Yes) × household income(=$200,000 and above)	0.76(0.67, 0.84)	< 0.0001	0.73(0.64, 0.83)	< 0.0001

Compared to White respondents, vaccination among Black respondents was associated with an additional 23% higher odds of anxiety (AOR = 1.23, 95CI% 1.12–1.35), and among Hispanic respondents was associated with an additional 15% higher odds of anxiety (AOR = 1.15, 95CI% 1.05–1.26) and 27% higher odds of depression (AOR = 1.27, 95CI% 1.15–1.39). These positive associations were stronger in people with lower educational background (AORs > 1, *p* < 0.05) (Table 2).

No gender disparities were found (Table 2).

The state-specific analysis indicated that the above association and subgroup disparities have obvious geographic variance (Supplement).

## Discussion

Here we demonstrated that being vaccinated for SARS-CoV-2 was associated with lower odds of anxiety and/or depressive symptoms, and this association varied among states. While those more middle-aged or more affluent, were more likely to show these negative associations, the contrary was observed in ethnic minorities and those with lower educational attainment.

Our hypotheses regarding the association between SARS-CoV-2 vaccination and reduced symptoms of anxiety and/or depression were confirmed by our analysis (Table 2). Although we don't have corresponding variables to enable us to verify the possible mechanism, it's easy to understand that the SARS-CoV-2 vaccination reduced people's fear of COVID-19 or related constrain resulting from COVID-19 controlling, and then reduced vaccinees’ mental problems. The supplement result indicated an obvious variation of the above association among states. This variation to some extent keeps in line with the variation of confirmed COVID-19 cases and different anti-COVID-19 measures took by states (Hallas et al., 2020), and also keeps in line with the anxiety-related events which only reported in part of vaccination sites in the US (Hause et al., 2021).

Older people and people widowed/divorced/separated have a stronger association between SARS-CoV-2 vaccination and reduced symptoms of anxiety and/or depression (Table 2). These results are consistent with previous studies, which concluded that these groups are more vulnerable to COVID-19 and are more likely to express willingness to be vaccinated against COVID-19 (Geriatric Medicine Research et al., 2021; Fisher et al., 2020; Wu et al., 2021; Holingue et al., 2020).

No gender disparity was found on the association between SARS-CoV-2 vaccination and reduced symptoms of anxiety and/or depression (Table 2), although females were found to suffer greater psychological distress during the COVID-19 pandemic (Holingue et al., 2020). This result also to some extent consistent with previous studies which concluded that females face additional stress sources than males (Wade et al., 2021; Hong et al., 2021), likes the difficulty to separate their work and family lives due to the pressure of traditional gender roles during the COVID-19 pandemic, and part of these additional stress source cannot be alleviated by reducing the fear on SARS-CoV-2.

Interestingly, although low-income people suffered more psychological distress from the COVID-19 (Holingue et al., 2020; McGinty et al., 2020), higher-income people benefit more from the association between SARS-CoV-2 vaccination and reduced symptoms of anxiety and/or depression (Table 2). In addition, vaccination among ethnic minorities and those with lower educational attainment associated with additional higher odds of anxiety or depression (Table 2). We suspect all of these could be an incidental effect arising from vaccine hesitancy that is more prevalent in these group people (Khubchandani et al., 2021; Fisher et al., 2020), and equally, unclarity and/or misinformation surrounding vaccination side effects (Hause et al., 2021; Hotez et al., 2021).

Anxiety-related events can occur after any vaccination (Hause et al., 2021). It is important for vaccination providers to aware that anxiety-related adverse events might occur if vaccinees unclear the possible adverse reactions, especially now that misinformation about the safety and effectiveness of vaccines is circulating everywhere (Hotez et al., 2021). Besides the recommended minimum of 15 min for observation of any adverse reactions after vaccination (Shimabukuro and Nair, 2021), more strategic and demography-sensitive public health communications for post-15 min are also needed.

The state-specific analysis presents further detailed information on which group of people should be focused on (Supplement). For instance, although there is no statistical difference at the national level, females in California and Michigan associated with additional higher odds of anxiety or depression after SARS-CoV-2 vaccination. The educational subgroup disparity mainly occurred in Lowa, Massachusetts, Minnesota, New Hampshire, Ohio, Pennsylvania, Texas, Vermont, and Wisconsin. The race/ethnicity subgroup disparity mainly occurred in Alabama, Connecticut, Georgia, Lowa, Louisiana, Nevada, New Jersey, North Carolina, Oklahoma, South Dakota, Texas, Vermont, Virginia, Wisconsin, and Wyoming. Although at the national level affluent people benefit more from the association between SARS-CoV-2 vaccination and reduced symptoms of anxiety and/or depression, the situation reversed in states including Louisiana and Rhode Island.

### Strength and limitations

To our knowledge, this is the first study to systemically assess the mental health outcomes after SARS-CoV-2 vaccination using national representative data. This study contributes to our understanding of the role of SARS-CoV-2 vaccination as a protective factor for the reduction of anxiety and/or depression. The subgroup analysis allowed more nuanced and practical public health policy strategies.

A key study limitation is the cross-sectional design and lack of pre-vaccination measures for the vaccinated cohort, precluding any causal inference i.e. is vaccination leading to the lower likelihood of anxiety or, were those less anxious and/or depressed more likely to get vaccinated in the first place. Other limitations include possible confounders unaccounted for (e.g. psychiatric and non-psychiatric comorbidities), and the lack of clinical confirmation as data was drawn from large-scale population survey using self-administered instruments. Finally, dataset limitation also hindered sensitivity testing of findings against lifestyle variables e.g. life stressors that could potentially explain correlations observed.

## Conclusion

Being vaccinated for SARS-CoV-2 was associated with lower odds of anxiety and/or depressive symptoms, but existed subgroup disparities to the disadvantage of ethnic minorities and those with lower educational attainment. More strategic and demography-sensitive public health communications could perhaps temper these issues.

## Role of the funder/sponsor

The funder had no role in the design and conduct of the study; collection, management, analysis, and interpretation of the data; preparation, review, or approval of the manuscript; or decision to submit the manuscript for publication.

## Reproducible research statement

Study protocol and statistical code: Available from Dr. Chen. Data set: available from https://www.census.gov/programs-surveys/household-pulse-survey/datasets.html mmc1.docx

## CRediT authorship contribution statement

**Shanquan Chen:** Data curation, Formal analysis, Visualization, Funding acquisition, Writing – original draft, Supervision, Software. **Athina R. Aruldass:** Funding acquisition, Formal analysis, Data curation, Writing – original draft, Supervision, Software. **Rudolf N. Cardinal:** Writing – original draft, Supervision.

## Declaration of Competing Interest

None reported.

## Appendix Supplementary materials

Image, application 1
