# Supplementary material for: Mental health outcomes after SARS-CoV-2 vaccination in the United States: A national cross-sectional study
Source: J Affect Disord. 2022 Feb 1;298(Pt A):396–9. doi: 10.1016/j.jad.2021.10.134 (PMC8580571; doi:10.1016/j.jad.2021.10.134)

**Sup figure 1. Association between SARS-CoV-2 vaccination and anxiety or depressive disorders by states.** The figure shows logistic regressions of the outcome in the subtitles on the taking of SARS-CoV-2 vaccination; horizontal lines represent 95% statistical confidence intervals. Covariates controlled for included age, gender, race/ethnicity, educational attainment, marital status, household income, history of COVID-19 infection, week of the interview, and state of origin.


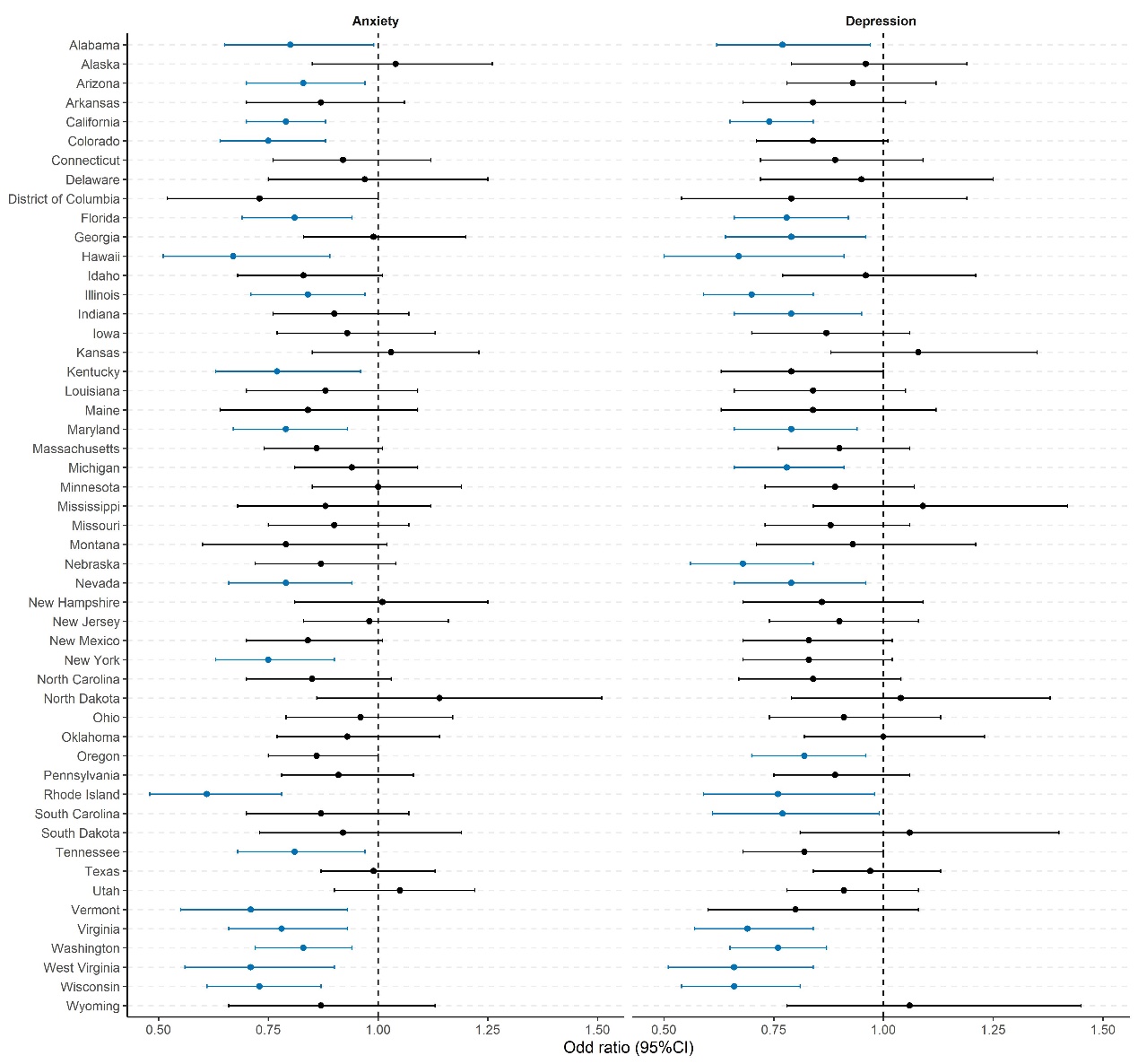


**Sup figure 2. Association between SARS-CoV-2 vaccination and anxiety or depressive disorders by states and age.** The figure shows logistic regressions of the outcome in the subtitles on the interaction of taking of SARS-CoV-2 vaccination and age (with 18-44 as reference); horizontal lines represent 95% statistical confidence intervals. Covariates controlled for included age, taking of SARS-CoV-2 vaccination, gender, race/ethnicity, educational attainment, marital status, household income, history of COVID-19 infection, week of the interview, and state of origin.


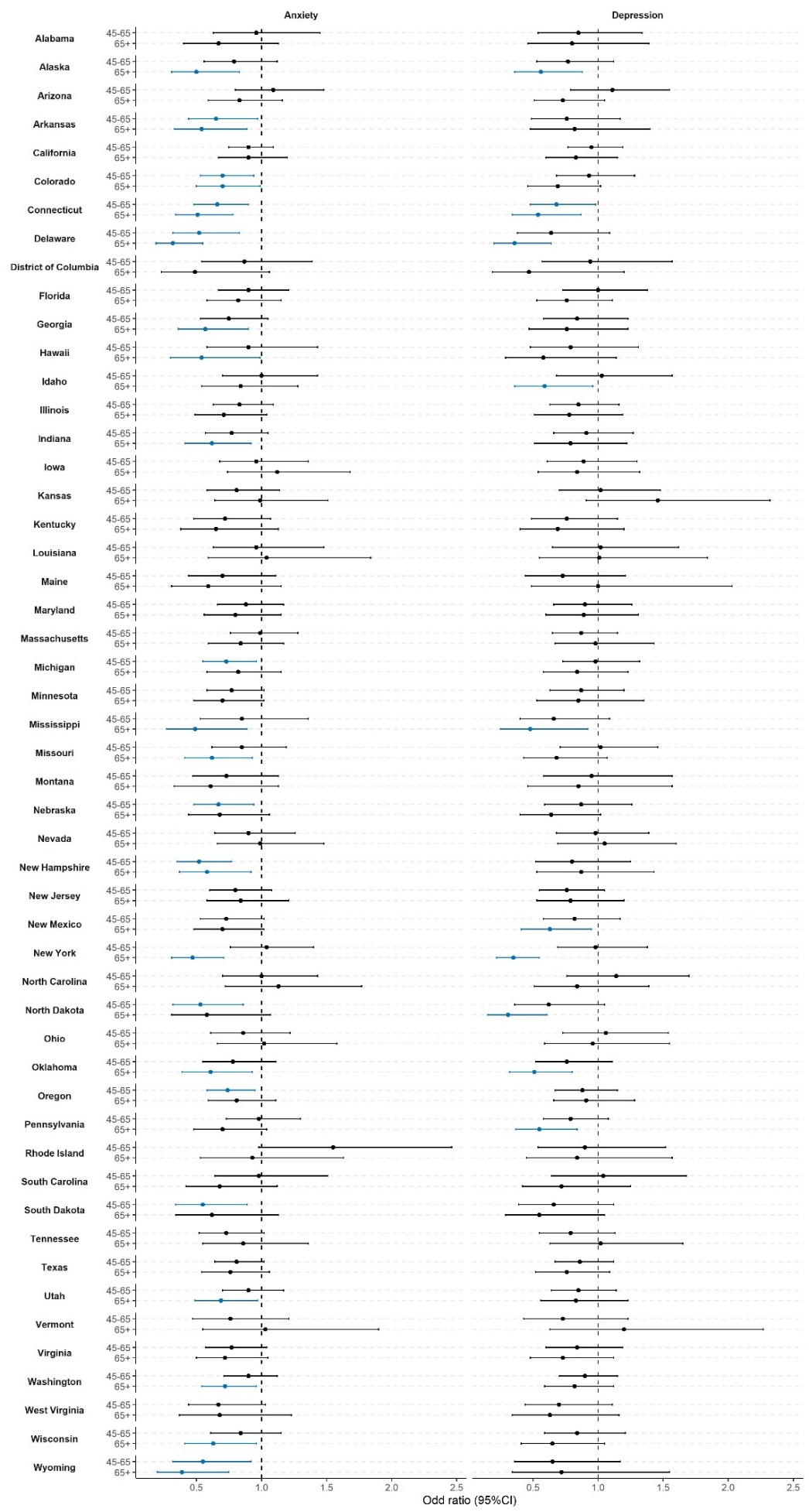


**Sup figure 3. Association between SARS-CoV-2 vaccination and anxiety or depressive disorders by states and gender.** The figure shows logistic regressions of the outcome in the subtitles on the interaction of taking of SARS-CoV-2 vaccination and gender (with male as reference); horizontal lines represent 95% statistical confidence intervals. Covariates controlled for included age, taking of SARS-CoV-2 vaccination, gender, race/ethnicity, educational attainment, marital status, household income, history of COVID-19 infection, week of the interview, and state of origin.


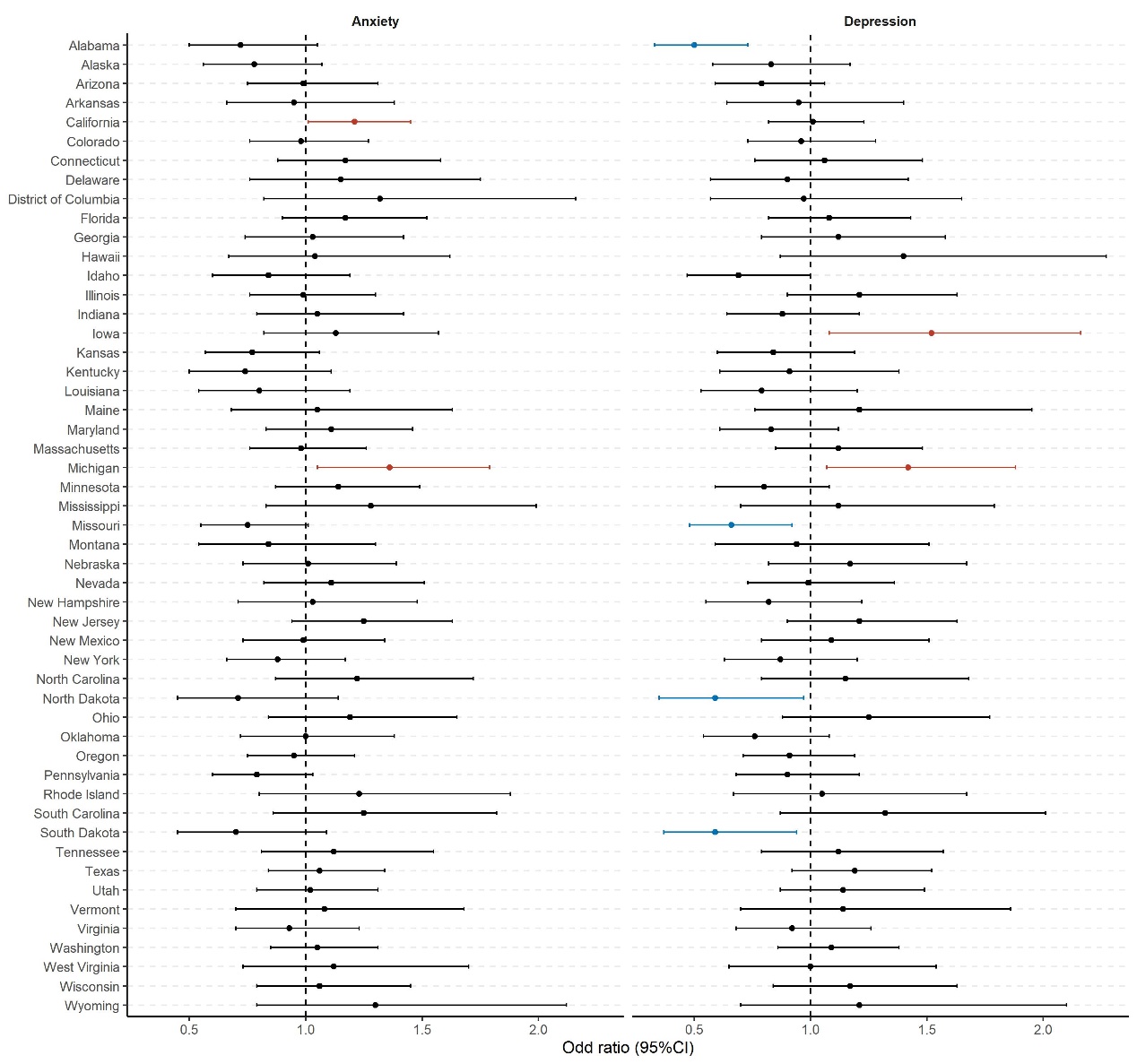


**Sup figure 4. Association between SARS-CoV-2 vaccination and anxiety or depressive disorders by states and racial status.** The figure shows logistic regressions of the outcome in the subtitles on the interaction of taking of SARS-CoV-2 vaccination and racial status (with White as reference); horizontal lines represent 95% statistical confidence intervals. Covariates controlled for included age, taking of SARS-CoV-2 vaccination, gender, race/ethnicity, educational attainment, marital status, household income, history of COVID-19 infection, week of the interview, and state of origin.


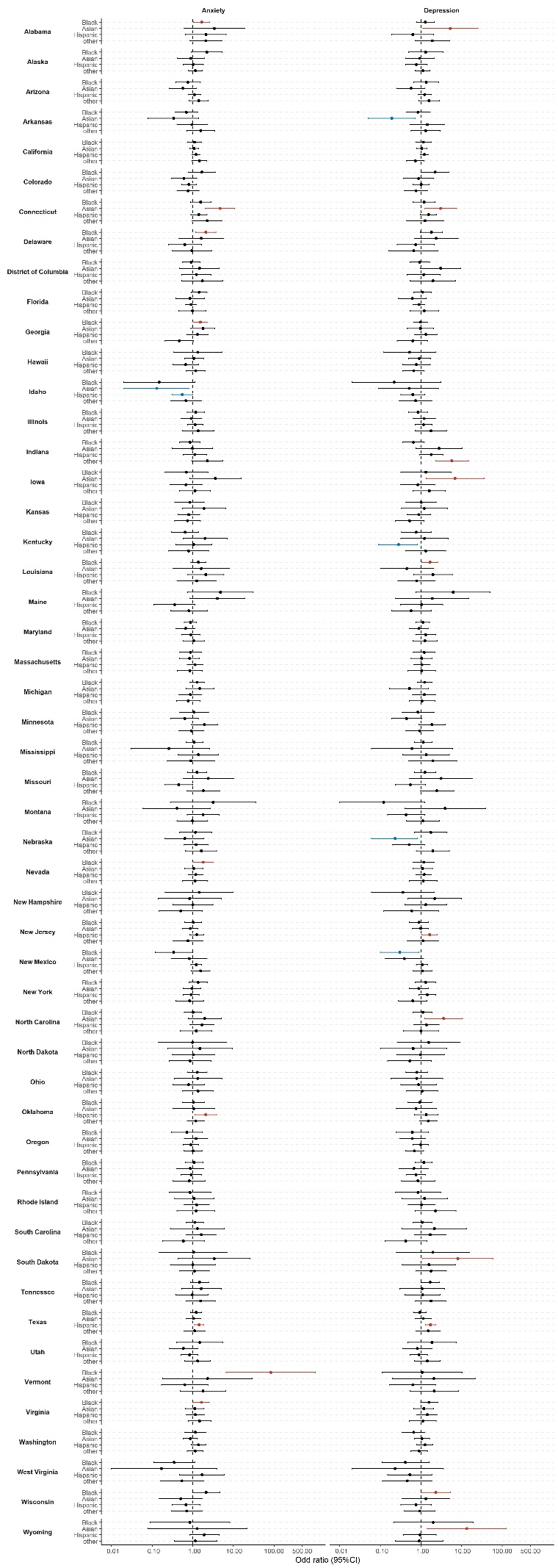


**Sup figure 5. Association between SARS-CoV-2 vaccination and anxiety or depressive disorders by states and marital status.** The figure shows logistic regressions of the outcome in the subtitles on the interaction of taking of SARS-CoV-2 vaccination and marital status (with married as reference); horizontal lines represent 95% statistical confidence intervals. Covariates controlled for included age, taking of SARS-CoV-2 vaccination, gender, race/ethnicity, educational attainment, marital status, household income, history of COVID-19 infection, week of the interview, and state of origin.


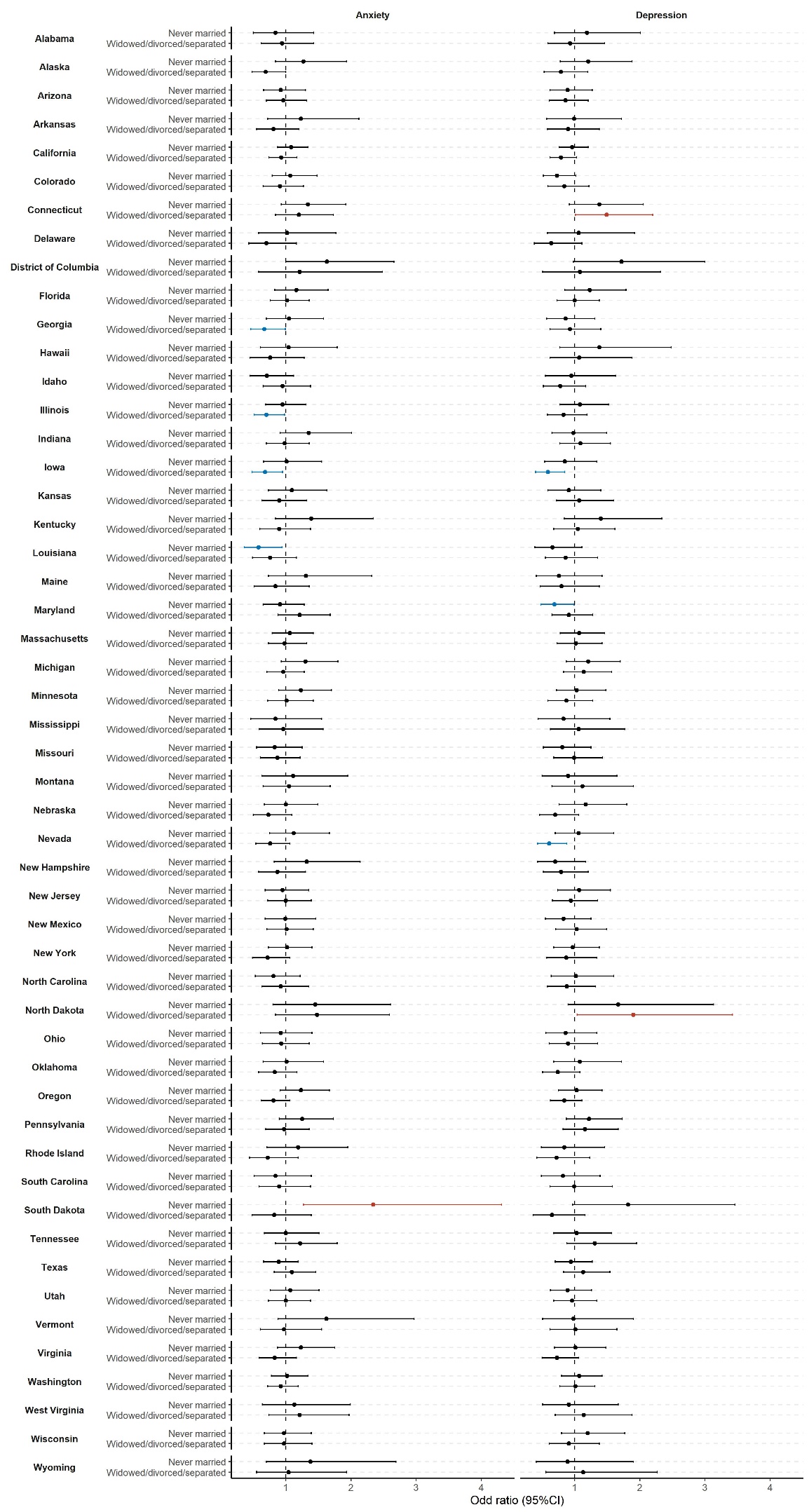


**Sup figure 6. Association between SARS-CoV-2 vaccination and anxiety or depressive disorders by states and household income.** The figure shows logistic regressions of the outcome in the subtitles on the interaction of taking of SARS-CoV-2 vaccination and household income (with less than $25,000 as reference); horizontal lines represent 95% statistical confidence intervals. Covariates controlled for included age, taking of SARS-CoV-2 vaccination, gender, race/ethnicity, educational attainment, marital status, household income, history of COVID-19 infection, week of the interview, and state of origin.


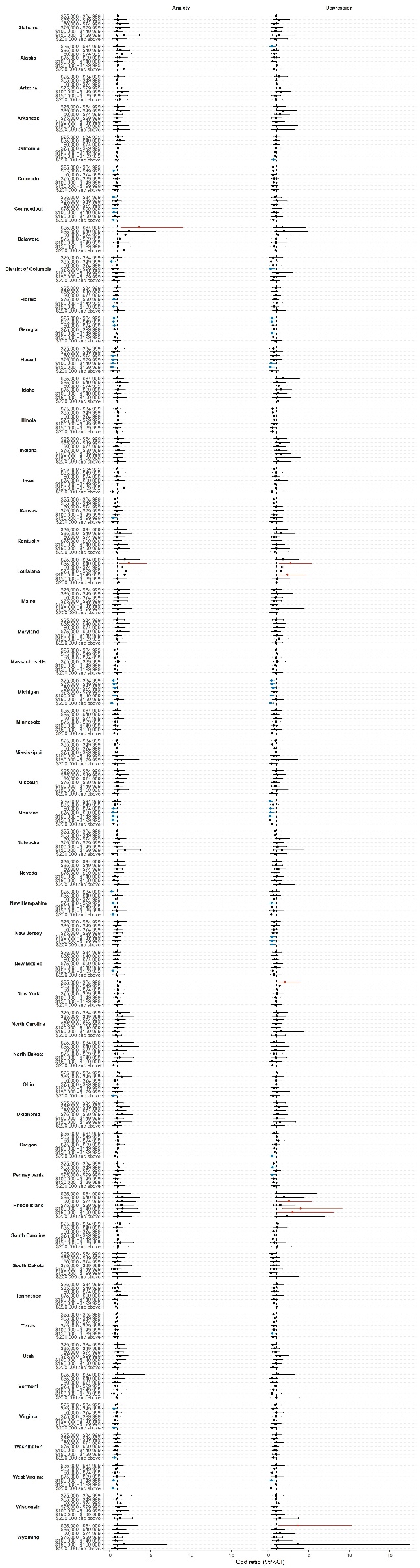


**Sup figure 7. Association between SARS-CoV-2 vaccination and anxiety or depressive disorders by states and educational attainment.** The figure shows logistic regressions of the outcome in the subtitles on the interaction of taking of SARS-CoV-2 vaccination and educational attainment (with graduate degree as reference); horizontal lines represent 95% statistical confidence intervals. Covariates controlled for included age, taking of SARS-CoV-2 vaccination, gender, race/ethnicity, educational attainment, marital status, household income, history of COVID-19 infection, week of the interview, and state of origin.


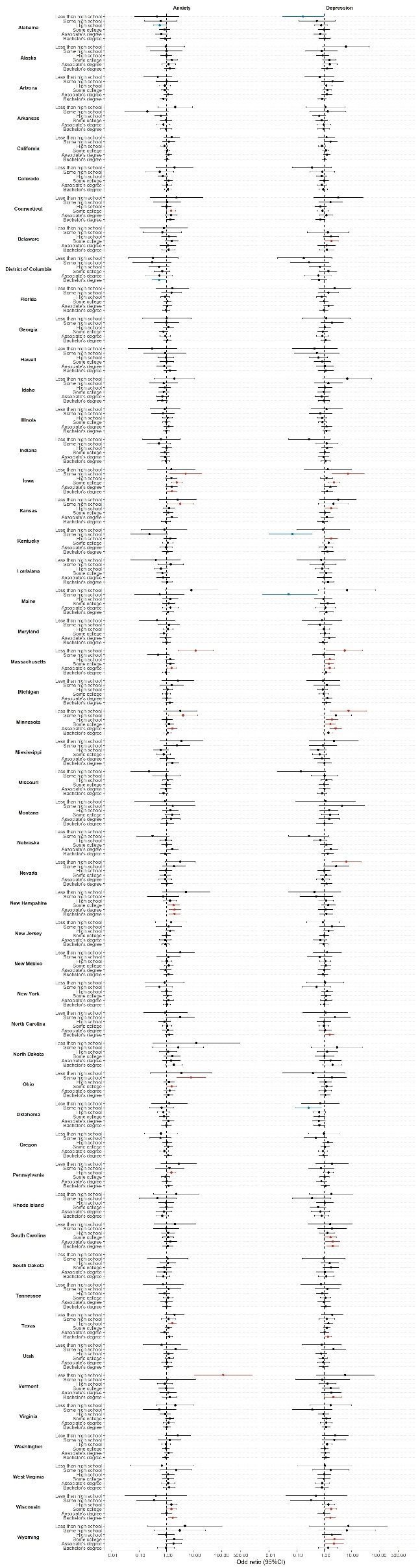

Supplement: Supplementary file 1 [file mmc1.docx]
